# Prevalence and Correlates of Helminth Co-infection in Kenyan HIV-1 Infected Adults

**DOI:** 10.1371/journal.pntd.0000644

**Published:** 2010-03-30

**Authors:** Judd L. Walson, Barclay T. Stewart, Laura Sangaré, Loice W. Mbogo, Phelgona A. Otieno, Benjamin K. S. Piper, Barbra A. Richardson, Grace John-Stewart

**Affiliations:** 1 Department of Global Health, University of Washington, Seattle, Washington, United States of America; 2 Department of Medicine, Division of Allergy and Infectious Disease, University of Washington, Seattle, Washington, United States of America; 3 Department of Pediatrics, University of Washington, Seattle, Washington, United States of America; 4 Department of Epidemiology, University of Washington, Seattle, Washington, United States of America; 5 Medical University of South Carolina, Charleston, South Carolina, United States of America; 6 Centre for Clinical Research, Kenya Medical Research Institute, Nairobi, Kenya; 7 Department of Biostatistics, University of Washington, Seattle, Washington, United States of America; University of Maryland School of Medicine, United States of America

## Abstract

**Background:**

Deworming HIV-1 infected individuals may delay HIV-1 disease progression. It is important to determine the prevalence and correlates of HIV-1/helminth co-infection in helminth-endemic areas.

**Methods:**

HIV-1 infected individuals (CD4>250 cells/ul) were screened for helminth infection at ten sites in Kenya. Prevalence and correlates of helminth infection were determined. A subset of individuals with soil-transmitted helminth infection was re-evaluated 12 weeks following albendazole therapy.

**Results:**

Of 1,541 HIV-1 seropositive individuals screened, 298 (19.3%) had detectable helminth infections. Among individuals with helminth infection, hookworm species were the most prevalent (56.3%), followed by *Ascaris lumbricoides* (17.1%), *Trichuris trichiura* (8.7%), *Schistosoma mansoni* (7.1%), and *Stongyloides stercoralis* (1.3%). Infection with multiple species occurred in 9.4% of infections. After CD4 count was controlled for, rural residence (RR 1.40, 95% CI: 1.08–1.81), having no education (RR 1.57, 95% CI: 1.07–2.30), and higher CD4 count (RR 1.36, 95% CI: 1.07–1.73) remained independently associated with risk of helminth infection. Twelve weeks following treatment with albendazole, 32% of helminth-infected individuals had detectable helminths on examination. Residence, education, and CD4 count were not associated with persistent helminth infection.

**Conclusions:**

Among HIV-1 seropositive adults with CD4 counts above 250 cells/mm^3^ in Kenya, traditional risk factors for helminth infection, including rural residence and lack of education, were associated with co-infection, while lower CD4 counts were not.

**Trial Registration:**

ClinicalTrials.gov NCT00130910

## Author Summary

Over one-third of people worldwide are currently infected with parasitic worms. The majority of these infections occur in sub-Saharan Africa, where over half of the population may be infected with at least one type of parasitic worm. HIV infection is also common in many of these countries, and there is significant geographic overlap in the presence of HIV and worm infection. Several studies have suggested that treatment of worm infections in people with HIV may delay the progression of HIV disease. Treatment has been shown to both decrease levels of the HIV virus in the blood of people with HIV and to increase the number of immune cells (CD4 cells) targeted by HIV. It is important to determine which populations of HIV-infected individuals are at greatest risk of worm infection in order to develop potential interventions for the treatment and prevention of worm infection in HIV-infected individuals. We report findings from a large study examining the prevalence and associated co-factors for worm infection among individuals at ten sites in Kenya.

## Introduction

Worldwide, more than 2 billion people are infected with at least one helminth species.[1] The majority of these infections occur in resource-limited settings, where over half of the population may harbor infection.[2] Given the significant geographic overlap of HIV-1 and helminth infections, a large proportion of HIV-1 infected individuals are likely to be co-infected with at least one helminth species.[3], [4] A pooled analysis of trials of deworming in HIV-1 infected individuals suggests significant benefit of deworming on both CD4 counts and plasma viral load.[5] Deworming has been estimated to reduce HIV-1 RNA by as much as 0.50 log_10_ copies/ml.[3], [4], [6] Modeling studies suggest that this magnitude of effect could delay HIV-1 disease progression by up to 25% and delay time to the development of AIDS by as much as 3.5 years.[7], [8] Despite HIV-1 prevalence rates exceeding 10–20% in some countries, widespread helminth prevalence surveys among HIV-1 infected individuals have not been conducted.[9] Because deworming may be a useful intervention in HIV-1 treatment programs, it is important to determine burden of helminth infection and cofactors for helminth infection in HIV-1 infected adults. However, there are limited surveillance data on helminth prevalence in HIV-1 infected adults in diverse geographic settings.

**Figure 1:**
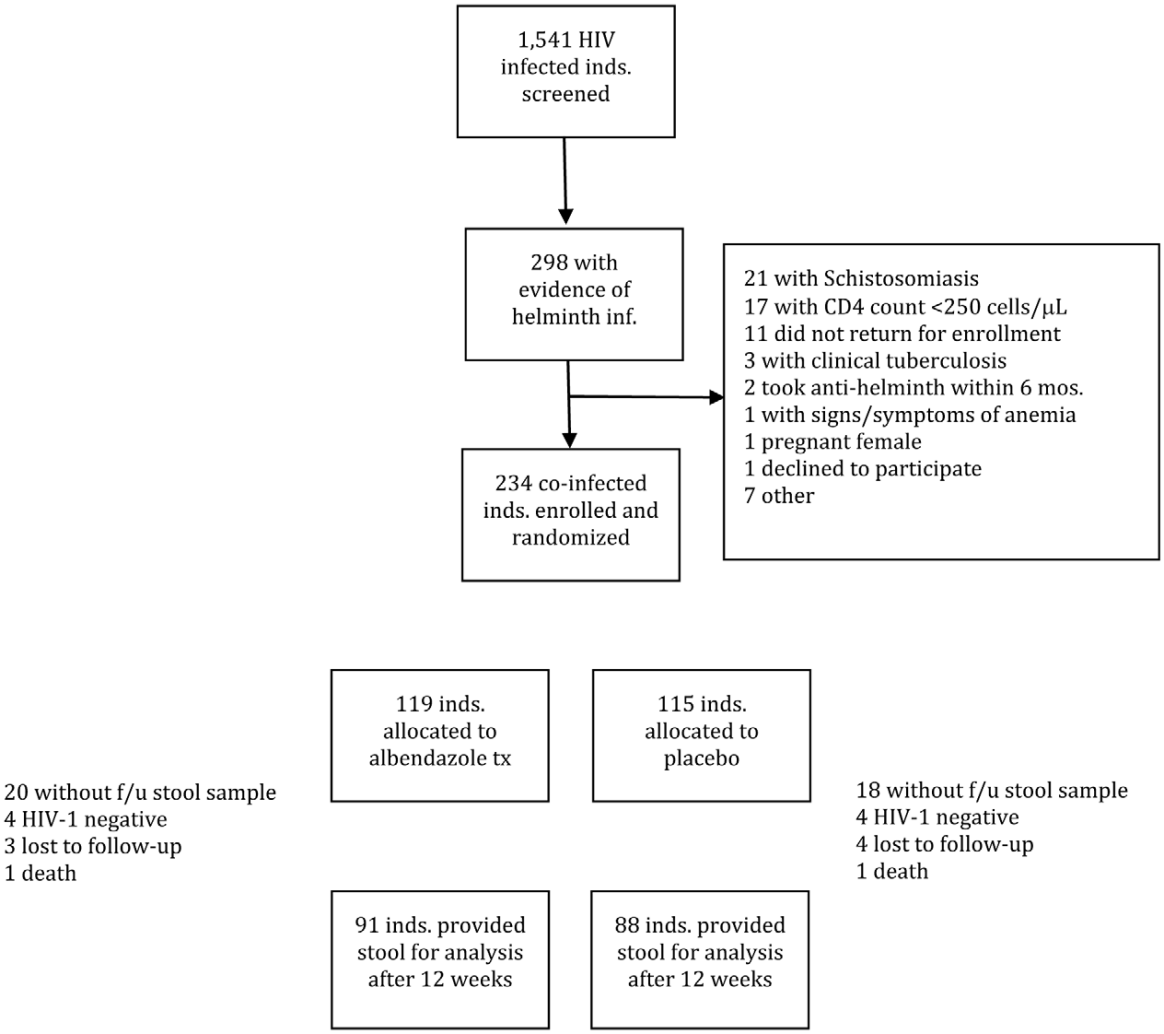
CONSORT flowchart of study screening and enrollment.

As part of a randomized trial of deworming, we determined prevalence and correlates of helminth infection among 1,541 HIV-1 infected adults attending ten geographically distinct HIV Care and Treatment Clinics in Kenya. We also determined factors associated with soil-transmitted helminth clearance and persistence/re-infection among 91 individuals who received albendazole as part of the clinical trial (Figure 1).

## Methods

### Study Design

**Figure 2:**
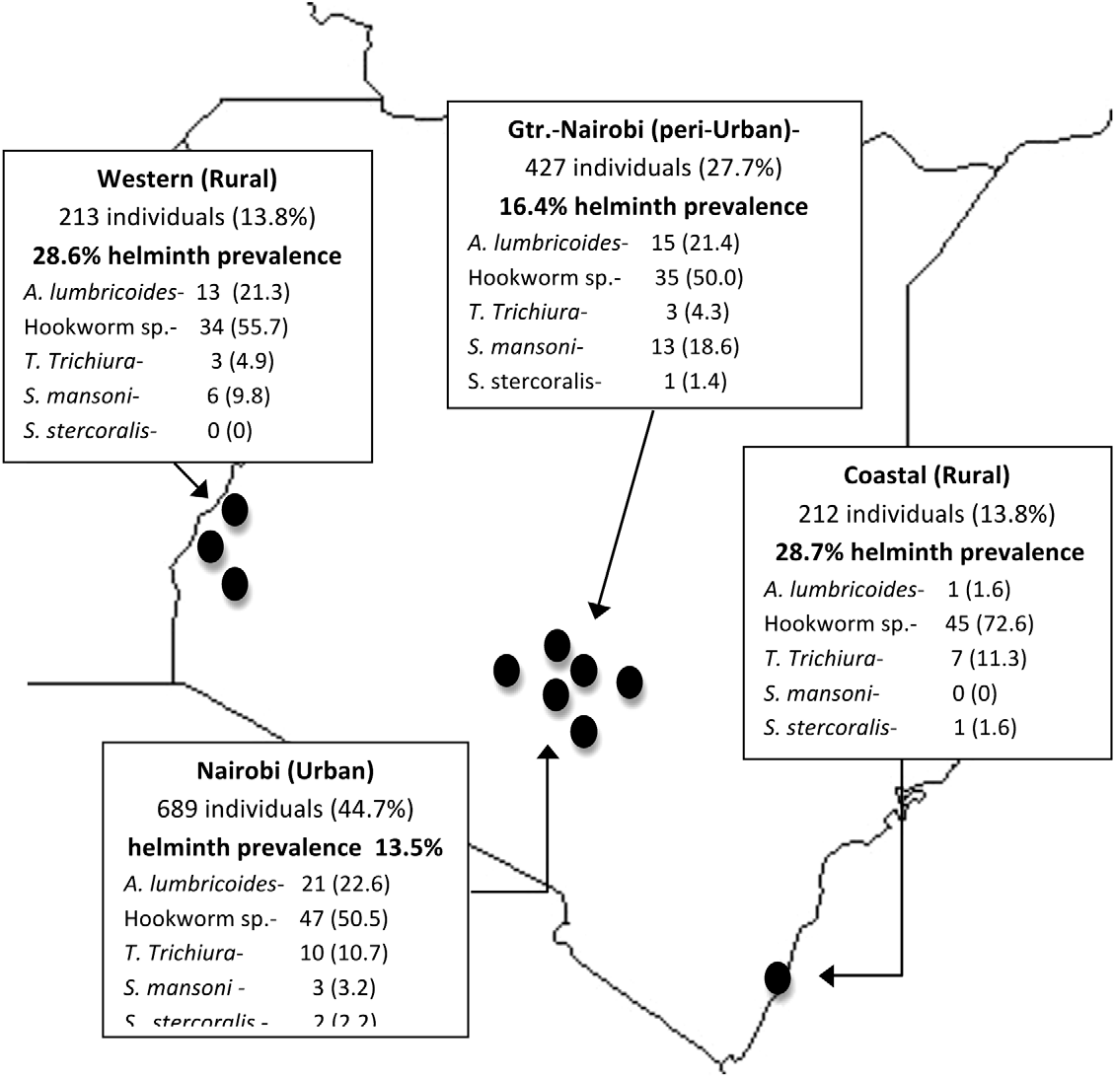
Geographic distribution and prevalence of helminth infection by species and screening region in Kenya. The black dots represent the relative locations of the ten sites where screening occurred. The number of individuals screened (and percent of total) in each region is listed. The prevalence of helminth infection in each region is listed below in bold. The number of infections for each species and column percents are shown for each region.

Antiretroviral-naïve HIV-1 seropositive adults not meeting WHO criteria for HAART initiation were screened by stool microscopy. Study participants were recruited from existing HIV Care and Treatment programs at ten sites geographically dispersed throughout Kenya using a mobile study team. Study sites were District Hospitals in Homa Bay, Kerugoya, Mbagathi, Thika, Kisumu, Kisii, Machakos and Kilifi, as well as the Kibera AMREF/CDC Clinic and the Coptic Hope Clinic (Nairobi) (Figure 2). The study was approved by the Kenya Medical Research Institute Ethical Review Board and the University of Washington Institutional Review Board. All participants provided written informed consent. The study was registered under Clinical Trials Registration identifier: NCT00130910.

Potentially eligible study participants were identified at each site by clinic staff and referred for helminth screening. Patients were eligible for helminth screening if they were HIV-1 seropositive, at least 18 years of age, non-pregnant and not eligible for initiation of antiretroviral therapy based on World Health Organization guidelines (CD4 cell count <250 cells/µL, WHO Stage 4 and Stage 3, other than for treated tuberculosis). Some individuals were enrolled based on a CD4 count obtained in the preceding three months and were subsequently found to have a CD4 count less than 250 cells/mm^3^ from the sample collected at enrollment. Exclusion criteria included having ever used antiretroviral drugs, having taken medicine for helminth infection in the preceding six months, having evidence of active tuberculosis (TB), and having clinical signs of severe anemia. Potential participants were informed of the aims and procedures of the study and assessed for eligibility. Participants were provided with stool collection containers, instructions on specimen collection, and were requested to collect stool within six hours of the screening visit. All female participants were asked to provide a fresh urine sample for β-human chorionic gonadotropin testing to exclude pregnant women. Baseline demographic information and a medical history were obtained from participants at the time of screening.

Individuals with evidence of co-infection with albendazole-treatable soil-transmitted helminths (*A. lumbricoides*, *T. trichiura* or hookworm sp.) were enrolled into a randomized, double-blind, placebo controlled trial, details of which have been previously published.[3] Individuals with schistosomiasis or infection with *Taenia* species were treated with open-label praziquantel (and albendazole if co-infected with other helminths) and were not enrolled. Individuals with no evidence of helminth infection were counseled on basic hygiene and avoidance of helminth exposure, but were not enrolled in the randomized trial. Individuals who participated in the clinical trial were randomized to albendazole, (400 mg a day for three days) or placebo and followed for twelve weeks. CD4 counts and plasma HIV RNA assays were collected at baseline and at the twelve-week visit (Figure 1).

### Laboratory Analysis

Each participant provided a single stool sample separated into a plain collection vial (AlphaTec, California, USA) and a vial containing preservative (ProtofixCLR, AlphaTec). Each stool sample was processed within 6 hours of collection and read within 30 minutes of processing. Stool samples were evaluated using wet preparation, Kato-Katz and formol-ether concentration techniques by an experienced laboratory technician. The presence of protozoa or helminth eggs was recorded and the burden of infection, based on number of eggs per gram of stool, was calculated according to WHO standards.[10] Helminth infection was defined as the presence of ova or grossly visible helminth on any single examination.

All randomized participants underwent repeat HIV-1 serologic testing using Determine™ rapid test qualitative immunoassay (Abbott, Japan) and had CD4 counts and plasma viral loads determined at enrollment and follow-up. Randomized participants had CD4 counts measured at the time of enrollment using Multiset™ software on a FACSCalibur machine (Becton Dickinson, USA). Plasma HIV RNA was quantified on samples from randomized participants using the Gen-Probe HIV-1 viral load assay, which has been shown to quantify the subtypes of HIV-1 prevalent in Kenya.[11] CD4 count data from within the previous 3 months were abstracted from clinic records for participants who were screened for helminth infection but not enrolled in the randomized trial. No plasma viral load data were available for participants who were not enrolled in the randomized trial.

### Statistical Analysis

Analyses were performed using Stata version 9.2 (College Station, Texas, USA). All HIV-1 seropositive adults who were screened for helminth infection for the randomized clinical trial were included in these analyses.

Primary analyses identified correlates of helminth infection among HIV-1 infected adults, which included; age, gender, geographic area, education, employment, sanitation, water access, and CD4 count. Household development index, a composite measure combining education, sanitation, water source and occupation, was developed based on similar indexes in the literature.[12]–[14] The association between these factors and being infected with helminths at the time of screening was evaluated using relative risk regression.[15], [16] Multicollinearity was assessed and a multivariate model of the risk of infection with any helminth was developed using relative risk regression to assess the independent effects of the various correlates.

Chi-square tests were used to compare differences in the proportions of persistent or new infections at the 12 week follow-up visit after treatment with albendazole. Fisher's exact test was used if the expected value in any cell was less than 5. Comparisons of median CD4 counts and median log_10_ plasma HIV RNA between species were done using a Kruskal Wallis test. Stratified relative risk regression was used to determine the association between the correlates identified above and the risk of infection for each species. Because of the large number of analyses conducted for each species, only statistically significant results (p<0.05) are presented.

## Results

### Study Population

Between March 2006 and June 2007, stool from 1,541 HIV-1 infected antiretroviral-naïve adults at ten geographically distinct sites in Kenya was screened for evidence of helminth infection. The mean age of the participants was 35.5 years (range 18–80) and 74.5% percent were female. Most had less than a secondary school education (63%) and about half (51.5%) reported an income-generating occupation other than farming. Few individuals had access to piped water (12.3%), although most reported access to some form of latrine or toilet (93.6%). Individuals were recruited from urban clinics within Nairobi (44.7%), peri-urban clinics surrounding Nairobi (27.7%), rural clinics in western Kenya (13.8%) or a rural coastal clinic (13.8%). Based on eligibility criteria, individuals in this cohort were relatively immunocompetent (median CD4 count 410 cells/mm^3^, IQR 297-586). Median HIV-1 RNA was 5.0 log_10_ copies/mL, IQR: 4.3-5.4) in the subset of participants enrolled in the clinical trial.

A total of 298 (19.3%) individuals had evidence of infection with at least one species of helminth and 234 were enrolled into the randomized trial. Hookworm species was the most prevalent helminth identified, 56.4% (n = 168), followed by *Ascaris lumbricoides*, 17.1% (n = 51), *Trichuris trichiura*, 8.7% (n = 26), *Schistosoma mansoni*, 7.3% (n = 21), and *Stongyloides stercoralis*, 1.3% (n = 4). Mixed infection with at least two different helminth species was identified in 28 individuals (9.4%). Using WHO criteria, the majority of the infections were classified as being “light burden” with only 5 “moderate” and 2 “heavy burden” infections identified[10].

### Correlates of Helminth Co-infection

The geographic distribution of helminth infection in Kenya is displayed in Figure 2. Individuals in rural areas were more likely to be infected with at least one species of helminth compared to individuals in urban areas (RR 1.85, 95% CI 1.51–2.26).

Demographic characteristics including age, gender, education and occupation were evaluated as correlates for risk of helminth infection by univariate analysis (Table 1). There was a trend towards a lower risk of helminth infection with increasing age by decade (RR 0.88, 95% CI: 0.78–1.00). No statistically significant differences were found in the gender distribution between helminth infected and uninfected individuals. Lack of education was associated with an increased risk of helminth infection. Compared to individuals who completed secondary education, individuals with a primary school education were 29% more likely to be infected with any helminth (RR 1.29, 95% CI 0.99–1.68) and those with no education had nearly twice the risk of infection (RR 1.90, 95% CI 1.34–2.69). There was an increased risk of helminth infection among individuals who reported farming as an occupation compared to those with a non-farming occupation (RR 1.59, 95% CI 1.22–2.06).

**Table 1:** Correlates of helminth infection among HIV-1 seropositive adults in Kenya.

Characteristic	n	Helminth Infected (n = 298)	Helminth Uninfected (n = 1243)	RR (95% CI)
Age (years); mean (SD)	1488	34.4 (9.5)	35.7 (9.3)	0.88 (0.78, 1.00)*
Gender; n (%)				
Male	382	63 (22.3)	319 (26.3)	Reference
Female	1114	220 (77.7)	894 (73.7)	1.20 (0.93, 1.54)
Site; n (%)				
Nairobi (urban)	689	101 (33.9)	588 (47.3)	Reference
Greater-Nairobi (peri-urban)	427	74 (24.8)	358 (28.4)	1.18 (0.90, 1.56)
Western (rural)	213	62 (20.8)	151 (12.1)	1.99 (1.51, 2.62)
Costal (rural)	212	61 (20.5)	151 (12.2)	1.96 (1.49, 2.59)
Education; n (%)				
None	132	38 (14.1)	94 (7.9)	1.90 (1.34, 2.69)
Primary	782	153 (56.7)	629 (53.2)	1.29 (0.99), 1.68)
Secondary	449	68 (25.2)	381 (32.2)	Reference
Post-secondary	89	11 (4.1)	78 (6.8)	0.82 (0.45, 1.48)
Water source; n (%)				
Piped water in home	182	21 (7.5)	161 (13.4)	Reference
Communal water source	800	141 (50.2)	659 (54.8)	1.52 (0.99, 2.35)
Lake, river or pool	239	67 (23.8)	172 (14.3)	2.43 (1.55, 3.81)
Other	263	52 (18.5)	211 (17.5)	1.71 (1.07, 2.74)
Sanitation; n (%)				
Access to flush toilet	296	34 (12.1)	262 (21.6)	Reference
No toilet or latrine	95	37 (13.2)	58 (4.8)	3.39 (2.26, 5.08)
Pit latrine for single home	491	112 (39.9)	379 (31.3)	1.99 (1.40, 2.83)
Shared pit latrine	610	98 (34.9)	512 (42.3)	1.40 (0.97, 2.01)
Employment; n (%)				
Employed, non-farmer	767	125 (44.3)	642 (53.2)	Reference
Employed, farmer	263	68 (24.1)	195 (16.1)	1.59 (1.22, 2.06)
Not employed	460	89 (31.6)	371 (30.7)	1.19 (0.93, 1.52)
CD4 count (cells/µL); n (%)				
0 – 349	372	81 (30.3)	291 (40.8)	Reference
≥350	608	186 (69.7)	422 (59.2)	1.40 (1.12, 1.76)

*: Comparisons by years in decades.

Lack of access to clean water and adequate sanitation were also predictors of helminth co-infection. Compared to individuals with access to piped water in their homes, individuals obtaining water from a lake, river or pond had almost 2.5-fold increased risk of helminth infection (RR 2.43, 95% CI 1.55–3.81). When compared to individuals with access to a flush toilet, individuals with a pit latrine were twice as likely to be co-infected with helminths (RR 1.99, 95% CI 1.40–2.83), and those with no latrine were >3-fold more likely to have a helminth infection (RR 3.39, 95% CI 2.26–5.08) (Table 1). Individuals with the poorest household development index (HDI) had more than 2.5 times the risk of having a soil-transmitted helminth infection (RR 2.58, 95% CI 1.17–5.70) when compared to individuals with the most resources (Table 2). However, HDI was not associated with risk of infection from *S. mansoni*.

**Table 2:** Risk of helminth infection by household development index (HDI).

HDI level^a^	Soil-transmitted helminth (N = 264)		*S. mansoni* (N = 21)	
	%	RR (95% CI)	%	RR (95% CI)
Level 1	2.3	Reference	4.8	Reference
Level 2	68.6	2.20 (1.01, 4.79)	76.2	1.31 (0.18, 9.74)
Level 3	29.2	2.58 (1.17, 5.70)	19.1	0.95 (0.11, 8.34)

a Household development levels are defined as: Level 1 (most developed) participant has access to a flush toilet in home, water piped into their house, an income generating occupation, and has completed, at least, secondary school; Level 2 participant lacks one, two or three of access to a flush toilet, water piped into their home, an income generating occupation, and a secondary education; Level 3 (least developed) participant lacks four of the above.

Baseline CD4 count was correlated with risk of helminth infection in this cohort. The study aimed to recruit pre-HAART individuals with CD4 counts greater than 250 cells. Participants were allowed to give CD4 count measurements from their prior HIV Care and Treatment Clinic visit if these fell within 3 months of the screening date. However, when these CD4 counts were confirmed, many people had lower CD4 counts than recorded by history. Eighty-nine participants had CD4 counts between 0–199 cells/µL^3^ and 256 participants had CD4 counts between 200–349 cells/µL^3^. Compared to individuals with CD4 counts less than 349 cells/µL, those with higher CD4 counts (≥350 cells/µL) were 40% more likely to be infected with any helminth (RR 1.40, 95% CI: 1.12–1.76) (Table 1). HIV-1 RNA levels were not available for individuals not enrolled in the randomized trial (those without helminth infection), precluding an analysis of HIV-1 RNA as a correlate of helminth infection.

In a multivariate model including CD4 count, rural residence, education, water source, sanitation and occupation, higher CD4 count (RR 1.36, 95% CI: 1.07–1.73), rural residence (RR 1.40, 95% CI: 1.08–1.81), and having no education (RR 1.57, 95% CI: 1.07–2.30) remained independently associated with risk of helminth infection.

### Species-Specific Analyses

Species specific analyses were performed on all individual species with the exception of *S. stercoralis* (due to the small sample size (n = 4)). Correlates associated with species-specific infections are presented in Table 3. Hookworm species accounted for 56% of all infections in this population, and correlates associated with hookworm infection were similar to the correlates associated with any helminth infection in the overall cohort, with the exception that hookworm infection was not associated with farming occupation (p>0.05). Farming occupation was associated with increased risk of infection with *A. lumbricoides* (RR 2.02, 95% CI: 1.05–3.88), *S. mansoni* (RR 8.62, 95% CI: 2.78–26.8), and multiple species (RR 2.82, 95% CI: 1.03–7.67) when compared to individuals employed in non-farming occupations (Table 3). In addition, individuals living in Western Kenya were more likely to be infected with *A. lumbricoides* (RR 2.10, 95% CI: 1.09–4.07) and *S. mansoni* (RR 7.53, 95% CI: 1.90–29.8) when compared to individuals living in urban Nairobi.

**Table 3:** Relative risk of species-specific helminth infection associated with study correlates among HIV-1 seropositive adults in Kenya, compared to helminth-uninfected individuals.

Correlate	Hookworm sp. (n = 168) RR (95% CI)	*A. lumbricoides* (n = 51) RR (95% CI)	*T. trichiura* (n = 26) RR (95% CI)	*S. mansoni* (n = 21) RR (95% CI)	Mixed (n = 28) RR (95% CI)
Rural vs. urban residence	2.40 (1.81–3.17)	*	*	*	2.63 (1.27–5.47)
Increasing age in decades	0.78 (0.66–0.94)	*	*	1.58 (1.08–2.30)	0.59 (0.36–0.97)
Gender	*	*	*	*	*
Greater-Nairobi vs. Nairobi	*	*	*	6.48 (1.84–22.8)	*
Western vs. Nairobi	2.36 (1.58–3.51)	2.10 (1.09–4.07)	*	7.53 (1.90–29.8)	*
Coastal vs. Nairobi	2.83 (1.95–4.09)	*	*	*	2.74 (1.12–6.70)
None vs. secondary education	2.22 (1.38–3.58)	*	2.92 (1.04–8.22)	*	7.82 (1.45–42.0)
Environmental vs. piped water	3.40 (1.61–7.16)	*	*	*	*
Communal vs. piped water	2.46 (1.22–4.98)	*	*	*	*
No toilet vs. flush	5.08 (2.90–8.90)	*	*	*	8.28 (2.13–32.2)
Pit latrine vs. flush	2.17 (1.30–3.66)	*	*	*	*
Farmer vs. non-farmer	*	2.02 (1.05–3.88)	*	8.62 (2.78–26.8)	2.82 (1.03–7.67)
CD4 0-349 vs. ≥350 cells/µL	1.50 (1.10–2.05)	*	*	*	*

*: Non-statistically significant relative risk.

**Figure 3:**
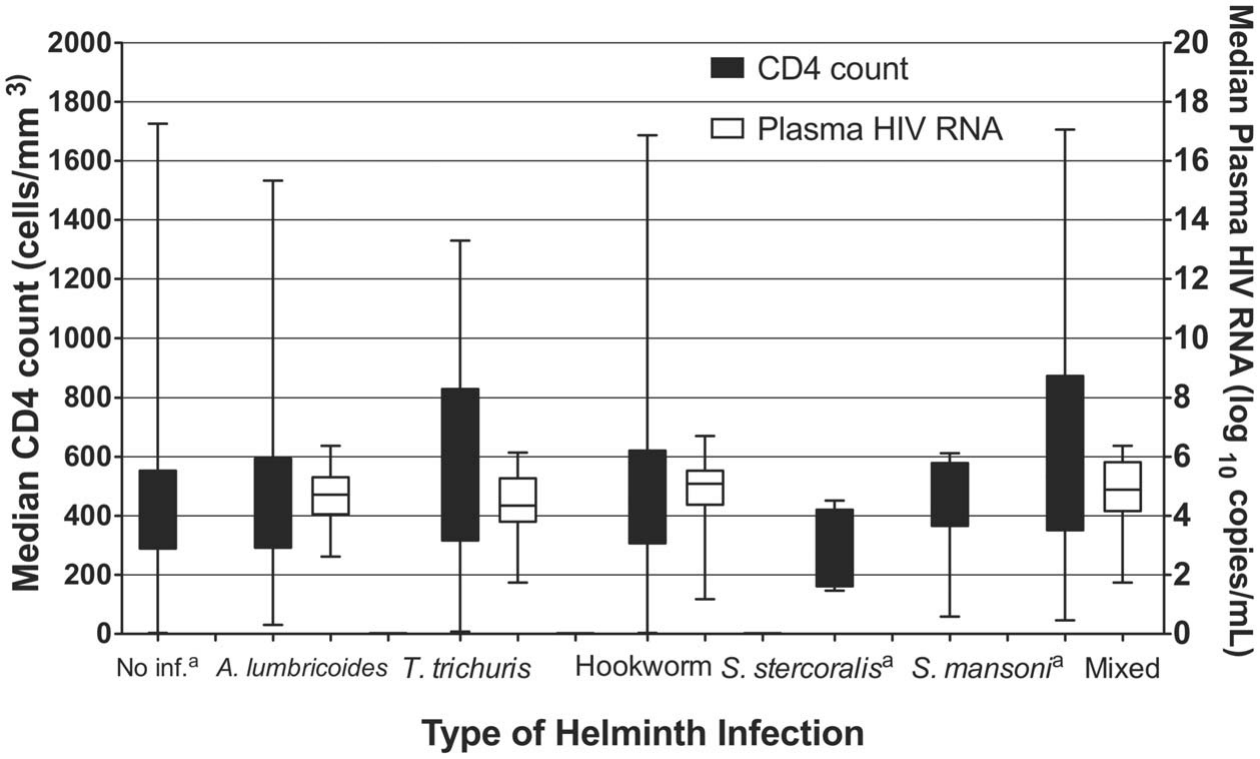
Median CD4 count and plasma HIV RNA level by helminth species. ^a^No HIV RNA data were available for *S. stercoralis* or *S. mansoni*. The study aimed to recruit pre-HAART individuals with CD4 counts greater than 250 cells. Participants were allowed to give CD4 count measurements from their prior HIV Care and Treatment Clinic visit if it fell within three months of the screening date. However, when these CD4 counts were confirmed, many people had lower CD4 counts than recorded by history. Eighty-nine participants had CD4 counts between 0–199 cells/µL^3^ and 256 participants had CD4 counts between 200–349 cells/µL^3^.

There were no differences in the median CD4 counts of individuals infected with different helminth species (p = 0.27) (Figure 3). Plasma log_10_ HIV RNA levels were similar between helminth species in the subset of individuals with available HIV-1 RNA levels (p = 0.10) (Figure 3).

### Risk of Persistent or Repeated Infection

**Figure 4:**
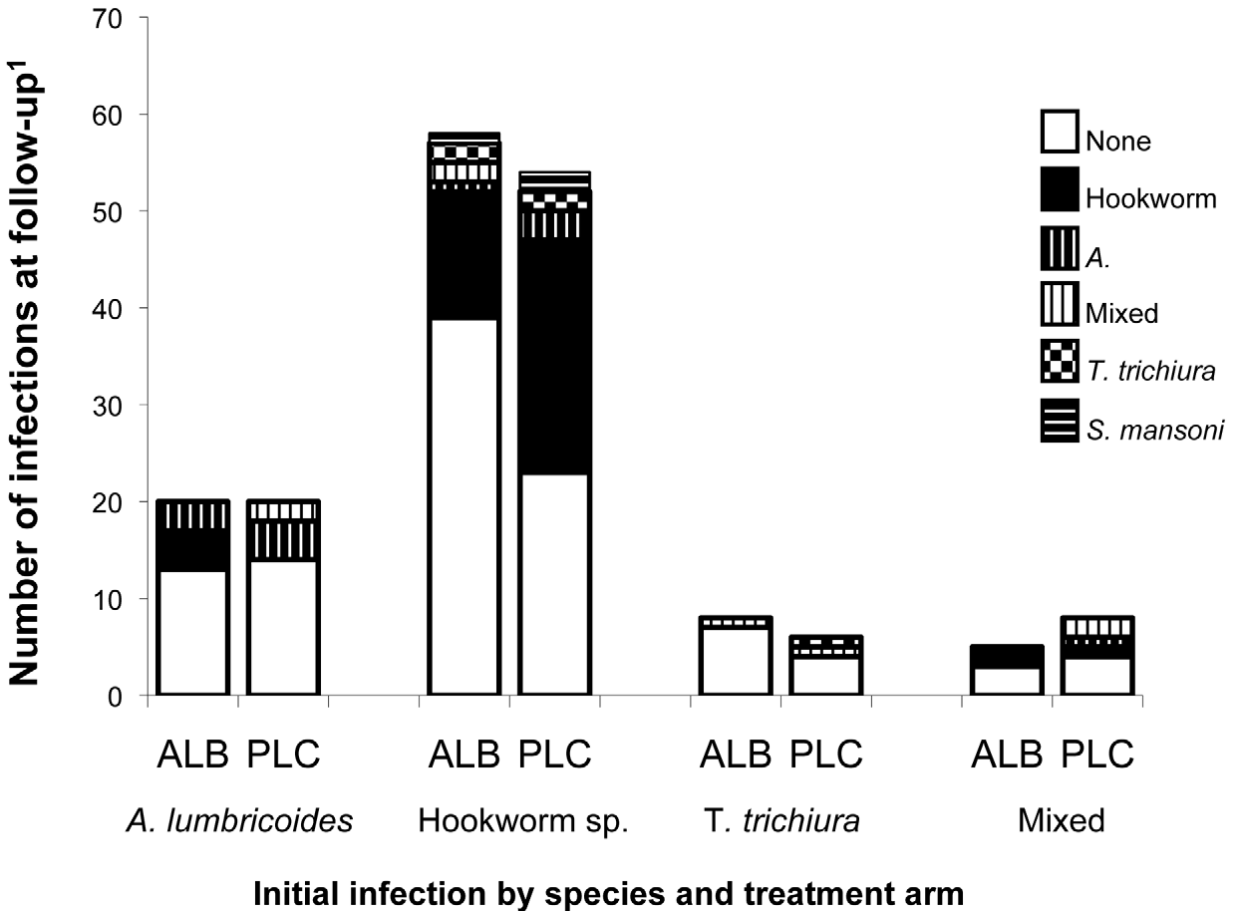
Species-specific helminth infection twelve weeks after albendazole or placebo treatment. ^1^Total number of individuals with follow-up stool available, stratified by species of initial infection and treatment arm. ALB, albendazole; PLC, placebo.

At the twelve-week follow-up visit, 179 individuals (76.5%) of the 234 individuals enrolled in the clinical trial provided stool for analysis (88 placebo arm vs. 91 treatment arm). Individuals in the treatment arm were significantly more likely to have evidence of cleared helminth infection at follow up than those in the placebo arm (68.1% vs. 51.1%, p = 0.02). Of those with evidence of helminth infection at follow-up (n = 72), 61.1% (44/72) had hookworm infection, 16.7% (12/72) had *A. lumbricoides*, 11.1% (8/72) had mixed infection, 6.9% (5/72) had *T. trichiura* and 4.2% (3/72) had S. mansoni infection. Baseline median CD4 counts were similar between those with infection at follow-up and those without (489 vs. 474, p = 0.99), as were baseline median log_10_ HIV RNA levels (5.1 log_10_ vs 4.9 log_10_ HIV RNA, p = 0.29). The distribution of initial and follow-up species-specific infections by treatment arm is displayed in Figure 4. There were no differences noted in the age, gender, educational level, occupation, access to sanitation or clean water between those who cleared their helminth infection and those who were infected at follow-up.

## Discussion

This study describes the prevalence and correlates of helminth co-infection among antiretroviral naïve, HIV-1 infected adults in Kenya. Helminth infection was common in HIV-1 infected adults in this study, with distinct geographic and sociodemographic risk factors for infection with different helminth species. Rural residence and education were independently associated with risk of helminth infection in this cohort and are similar to risk factors described in HIV uninfected individuals from similar settings. In addition, higher CD4 counts were also independently associated with risk of helminth infetion. In a smaller cohort of albendazole-treated individuals, helminths were detected relatively frequently 12 weeks after treatment, although region, CD4 count, and educational level were not associated with persistence/re-infection.

It has been estimated that more than a third of all individuals in sub-Saharan Africa are infected with at least one species of helminth, with considerable overlap in the prevalence of helminths and HIV-1.[17], [18] The prevalence and species distribution of helminth infection in our study were within the range of previously published reports of helminth infection in Africa, although lower than some previous estimates for Sub-Saharan Africa, where individual species prevalence has been estimated as 25% for Ascariasis, 29% for hookworm, and 24% for trichuriasis.[1], [17], [19], [20] The lower rates of infection in this cohort may reflect lower prevalence in older age groups and/or the use of a single stool specimen for diagnosis, which is less sensitive than the use of three samples used in many surveys.[21] A 24.9% prevalence of helminth infection was reported in 297 HIV-1 infected individuals in an urban setting in Zambia, which was higher than noted in population-based helminth surveys from the same urban population. However, in studies directly comparing helminth prevalence between HIV-1 infected and uninfected individuals in Africa, no consistent correlation between HIV-1 infection and increased risk of infection with any helminth species has been reported.[22]–[25] Previous studies have been limited by relatively small sample sizes (60-582 individuals) and have assessed helminth prevalence at a single site. Our study had a large sample size (1541 individuals) derived from geographically diverse urban and rural sites, providing fairly comprehensive assessment of helminth infection in HIV-1 infected adults in urban and rural throughout Kenya. Our study suggests that helminth prevalence in HIV-1 infected individuals is similar to general population prevalence in endemic settings.

In this cohort of HIV-1 infected adults in Kenya, rural residence, lack of education, poor sanitation, use of environmental sources of water and farming occupation were associated with helminth infection. However, in multivariate analyses, only education and rural residence remained predictive of risk of infection, suggesting that many of these cofactors share determinants. Poverty, lack of access to safe water and sanitation, agricultural exposure, household crowding, toileting practices, and education level are well described risk factors for infection with soil transmitted helminths.[26]–[29] Our observations differ from those reported in a previous study examining co-factors for helminth infection among HIV-1 infected adults in Lusaka, Zambia, which failed to find significant associations between water source, sanitation or occupation and helminth infection status.[30] The Zambian study was conducted in an urban setting, where sanitation, access to potable water and occupational exposure were likely different than the population reported in our study. In addition, the relatively small sample size included in the Zambian study (297 individuals) may have limited the power to detect associations. Our study suggests that correlates of helminth infection are similar among individuals with and without HIV-1 co-infection.

Helminth infected individuals in this cohort had significantly higher CD4 counts when compared to helminth uninfected individuals. It is possible that individuals with lower CD4 counts may be at lower risk of exposure, for instance by being less active and less likely to engage in agricultural work. There are conflicting data on the relationship between HIV-1 related immunosuppression and the acquisition of helminth infection.[31] Among HIV-1 infected pregnant women in Uganda, CD4 count was inversely associated with risk of hookworm infection (OR for hookworm infection per 100 cells/uL increase in CD4 count of 1.16 (95% CI 1.02–1.31)).[32] However, declining CD4 counts have been associated with higher burdens and greater severity of *Strongyloides* infection.[33] In addition, significant reductions in the prevalence of *A. lumbricoides*, *T. Trichiura*, hookworm sp., and *Strongyloides* have been reported following the introduction of highly active antiretroviral therapy (HAART), suggesting that HAART may result in improved immunologic protection and control of helminth infection.[33]–[40] In this study, only individuals with helminth infection were eligible to be enrolled in the randomized trial, and therefore would have had more recent CD4 data than helminth uninfected participants who were not eligible for the clinical trial. The resulting measurement bias would be expected to result in *lower* CD4 counts in those with helminth infection, not higher counts as observed. It is also important to note that the study included antiretroviral naïve individuals with CD4 counts greater than 250 cells/mm^3^ and without WHO Stage 3 or 4 disease, and therefore may not be representative of individuals with severe HIV-1 related immunosuppression or those on antiretroviral therapy. Despite these limitations, our study suggests that moderate immune suppression does not increase susceptibility to helminth acquisition.

The variable geographic distribution between different species of soil-transmitted helminths has important implications for treatment and control programs, particularly because the impact of helminth infection on HIV-1 may be species-specific.[41] We previously reported that albendazole treatment in *A. lumbricoides* co-infected individuals resulted in significant CD4 benefit but this effect was not seen for other helminths.[5] Previous studies also suggest that treatment of *A. lumbricoides*, *S. mansoni*, and lymphatic filiariasis may result in delaying HIV-1 disease progression, while treatment of *T. trichiura* and hookworm species may not.[3], [6], [42] These differences may be attributable to significant differences in the level of tissue invasiveness, antigen burden, duration of infection and host immune response between species.[42], [43] We found that approximately one-sixth (17.1%) of all helminth infections among HIV-1 infected adults in Kenya and 11.1% of detectable infections following albendazole treatment were due to *A. lumbricoides*. The prevalence of ascariasis likely reflects the age of the included population and is consistent with prior studies in the general population, which showed that *A. lumbricoides* prevalence peaks before age 10 and then decreases with age.[20], [44] The most prevalent species we detected was hookworm (56.4% of infections). This is higher than in population-based prevalence surveys in Kenya where hookworm accounts for 36-46% of infections, but consistent with reported higher prevalence rates of hookworm with increasing age[20], [45], [46] Despite suboptimal detection techniques for schistosomal infections in this cohort, *S. mansoni* was prevalent among individuals screened in western and central Kenya, where it has previously been reported to be endemic.[47]

Almost a third (32.1%) of the individuals receiving three doses of albendazole in this trial returned with helminth infection after 12 weeks. It is not clear whether the infections detected at follow-up represent treatment failures or repeat infections. While albendazole is not completely effective for the treatment of helminth infections, individuals also returned to the same home environments following treatment, suggesting continuous environmental exposure to infection risk. Our findings suggest that repeated treatment, in combination with health education programs to minimize exposure risk, may be needed to maximize the efficacy of deworming in HIV-1 infected individuals.

Strengths of this study include the screening of a large number of HIV-1 infected individuals from geographically distinct sites in Kenya for helminth infection, the use of a combination of screening techniques to increase the sensitivity of helminth diagnosis, and the systematic measurement of outcome measures with a randomized trial. However, this study also had limitations. While the combination screening methods used in this study are widely accepted, they have poor sensitivity for the detection of soil-transmitted helminth species.[48] The variability in sensitivity of stool diagnostic techniques is evident in the failure to detect helminth infection in half of individuals with previously detected helminths who received placebo. In addition, individuals were not screened with sensitive detection methods for schistosomiasis or strongyloidiasis. It is likely that we underestimated the true prevalence of helminth infection. This misclassification would have decreased the detection and the strength of reported associations. Approximately forty-five percent of study participants were recruited in Nairobi, which may have over-sampled individuals residing in urban populations. However, living conditions in areas of Nairobi (such as Kibera, Kerugoya or other urban slums) often include dirt floors, open sewage and unsafe water sources, all potential environmental sources of helminth infection. Finally, we excluded individuals with CD4<250 cells/mm^3^, which limited ability to determine effects of severe immunosuppression.

In summary, co-infection with HIV-1 and helminths is common among adults in Kenya and is associated with rural residence and lack of education. Moderate immunosuppression does not appear to increase the risk of helminth acquisition in HIV-1 infected adults. A combination of repeated deworming with anti-helminthics, as well as education on helminth-prevention, may be necessary to eradicate helminths in HIV-1 infected adults. Given the relatively high prevalence of helminth infection documented in this study and the available data suggesting possible benefit of deworming on markers of HIV-1 disease progression, measures to eradicate and prevent helminth infections may be a feasible public intervention that complements other interventions to delay immunosuppression in HIV-1 infected individuals.

## Supporting Information

Checklist S1 CONSORT checklist.
(0.19 MB DOC)

Protocol S1 Trial protocol.
(0.20 MB PDF)
