# Supplementary material for: Prevalence and Correlates of Helminth Co-infection in Kenyan HIV-1 Infected Adults
Source: PLoS Negl Trop Dis. 2010 Mar 30;4(3):e644. doi: 10.1371/journal.pntd.0000644 (PMC2846937; doi:10.1371/journal.pntd.0000644)
Supplement: Protocol S1 — Trial protocol. (0.20 MB PDF) [file pntd.0000644.s002.pdf]

Project Proposal for Kenya Medical Research Institute, Centre for Clinical Research

Title: Treatment of helminth co-infection: short-term effects on HIV-1 progression markers and immune activation

Investigators and Institutional Affiliations:

Fellow Investigator/University of Washington:
Judd L. Walson, MD, MPH
Senior Fellow in Infectious Diseases
Box 359909
325 Ninth Avenue
Seattle, WA 98104-2499
E-mail: walson@u.washington.edu
CV attached

Principal Investigator/Kenya Medical Research Institute
Phelgona Otieno, MBChB, MMed (Paeds), MPH(Epi)
Senior Medical Research Officer and Consultant Paediatrician
Kenya Medical Research Institute
Centre for Clinical Research
Mbagathi Way
PO Box 20778
Nairobi, Kenya

Principal Investigator/University of Washington
Grace John-Stewart, MD, PhD, MPH
Associate Professor
Department of Medicine
University of Washington
325 Ninth Avenue
Seattle, WA 98104-2499

Co-investigators

Margaret Mbuchi, PhD
Senior Research Officer
Kenya Medical Research Institute
Centre for Clinical Research
Mbagathi Way
PO Box 20778
Nairobi, Kenya

Frederick Kirui, MBBS
Assistant Research Officer
Kenya Medical Research Institute
Centre for Clinical Research
Mbagathi Way
PO Box 20778
Nairobi, Kenya

Michael Chung, MD, MPH
Acting Instructor
Department of Medicine
University of Washington
325 Ninth Avenue
Seattle, WA 98104-2499

Dr Eduard Sanders MD PhD
Centre for Geographic Medicine Research Coast
PO Box 230, Kilifi, 80108, Kenya
esanders@kilifi.kemri-wellcome.org

Dr James Berkley MB BS MTropMed MRCP MD
Centre for Geographic Medicine Research Coast
PO Box 230, Kilifi, 80108, Kenya
JBerkley@kilifi.kemri-wellcome.org

Barbara Lohman PhD
Acting Instructor
Department of Medicine
University of Washington
325 Ninth Avenue
Seattle, WA 98104-2499

Abstract:

Most HIV-1 infected individuals in areas of high seroprevalence live in crowded urban settings. These conditions are associated with repeated helminth infections, which may alter immune function increasing the susceptibility and severity of other infections in these individuals. Prior observational studies have shown that in HIV-1 infected individuals, helminth infections are associated with immune activation and increased HIV-1 RNA levels. It is important to determine whether treating helminth co-infection in patients with HIV-1 will slow disease progression or reduce transmission.

We plan to determine the prevalence of helminth infection in HIV infected adults, characterize the short-term effect of antihelminth therapy on HIV-1 disease progression and immune activation markers, and examine co-factors for helminth infection, such as CD4 count, viral load, age, gender and duration of time spent in an urban setting. In addition, we will conduct a randomized clinical trial to determine the effect of helminth eradication on markers of immune activation and HIV-1 disease progression and transmissibility. The study will be conducted in a group of HIV-1 infected individuals who do not yet meet criteria for HAART. Identifying inexpensive and practical methods to delay HIV-1 progression offers tremendous public health benefits. At the present time, highly active antiretroviral therapy is being rapidly scaled up in resource poor settings. The demand for these drugs currently far outweighs the capacity for such services to be provided in many regions. For the millions of people affected and infected by HIV-1 in resource poor settings, delaying immunosuppression for months to years could allow important developments in infrastructure that would permit the maximum number of individuals to benefit from highly active antiretroviral therapy. In addition, it is important to identify inexpensive and effective methods to reduce HIV-1 transmission.

Introduction and Background:

Of the approximately 25 million HIV-1 infected individuals currently living in Africa, as many as 50-90% may be co-infected with soil transmitted helminths such as roundworms, hookworms or whipworms.[1,2] Several studies examining the effect of helminth co-infection on individuals infected with HIV-1 suggest that these helminth co-infections may increase HIV-1 viral load and may lead to more rapid progression of HIV-1 disease.[3] In many resource-poor settings, HIV-1 treatment programs are being rapidly scaled up to provide antiretroviral therapy to immunosuppressed HIV-1 infected individuals. These programs offer significant potential benefit to the millions of infected individuals who currently meet treatment criteria. However, additional strategies must be developed in order to delay immunosuppression and antiretroviral initiation for the majority of infected individuals who do not yet meet criteria for treatment (CD4 count ≥200 cells/mm^3). Delaying immunosuppression among HIV-1 infected individuals promises to spare antiretroviral toxicity, conserve the limited repertoire of antiretroviral options, and to prolong survival. Co-infection prophylaxis merits evaluation because it may provide an effective and relatively inexpensive means to delay immunosuppression in these individuals.

Helminth infection leads to significant stimulation of the host immune response. These infections are often characterized by the daily production of millions of eggs, excretory products, and secretions that result in a state of persistent activation of the host immune system. Specifically, helminth infected individuals display increased levels of eosinophilia, increased IgE levels, and a Th2 immune bias.[4,5] The effects of such immune activation are not entirely clear, but several studies have suggested that this increased activation of the Th2 immune subset may further enhance HIV replication and progression.[6] In addition, the enhanced Th2 response seen in these infections may have a detrimental effect on the host's

ability to respond to other infections, particularly those dependent on Th1 immune responses, such as tuberculosis.[7]

In an Ethiopian study of HIV-1 infected and uninfected individuals, helminth infections resulted in increased memory CD4 cells and increased T-cell activation, and treatment of helminth infections was associated with decreased T-cell activation.[8] Interestingly, among the HIV-1 infected individuals in this observational study, treatment of helminth infection resulted in a significant increase in absolute CD4 counts (192 versus 279 cells/mm^3, p=0.002). In another study of 56 HIV-1 infected individuals in Ethiopia, 31 (55%) had helminth co-infections with one or more species detected in stool samples, mainly Ascaris lumbricoides and Trichuris trichiura.[9] At baseline, there was a strong association between stool helminth burden and plasma HIV-1 RNA levels among individuals with helminth co-infection ($p<0.001$). In addition, successful treatment of co-infection (as documented by clearance of helminth eggs in stool) led to a significant decrease in HIV-1 plasma viral load (-0.36 $\log_{10}$) in these patients. This change in viral load was significantly greater than that seen in those individuals without documented clearance of their helminth co-infection (+0.67 $\log_{10}$) (p=0.04).[10] Other studies suggest that this effect may be variable, depending both on the type and duration of helminth co-infection as well as on host immune status. In one such study from Kenya, plasma HIV-1 RNA levels did not decrease significantly following treatment of schistosomiasis.[11]

The majority of HIV-1 infected individuals do not yet meet criteria for HAART. These individuals continue to transmit the virus and perpetuate the epidemic. For each log increase in plasma HIV-1 viral load, there is an estimated 2.5-fold increased risk for transmission to the sexual partner of the infected individual.[12] Thus, the benefit of reducing viral load by even 0.5 log could be substantial. Identifying inexpensive and feasible interventions that may reduce viral load has the potential for significant public health benefit.

Justification of the Study:

To date, there are no randomized clinical trials evaluating the effect of anti-helminth treatment on immune activation, CD4 count, and HIV-1 viral levels. It is important to determine whether routine treatment of helminth co-infection should be considered standard practice in the treatment of HIV-1 infected individuals in helminth endemic areas. Such treatment may be a useful strategy to delay HIV-1 progression and prevent transmission of HIV-1 among individuals in resource-poor settings. Interval anti-helminth therapy may be a feasible option in many areas of the world to delay immunosuppression,,to enhance the response to antiretroviral therapy or to reduce infectiousness in HIV-1 infected individuals. In addition, as patients progress to HAART, it is important to determine the ideal timing for helminth eradication.

Hypothesis: Treatment of gastrointestinal helminths in patients with HIV-1 may impact markers of disease progression and infectiousness, namely CD4 count and plasma and gential HIV-1 RNA levels, when compared to no treatment in these individuals.

Overall General Objectives:

To evaluate the effect of helminth co-infection on HIV-1 disease progression and transmission in a cohort of HIV-1 infected Kenyan adults who do not meet criteria for highly active antiretroviral therapy.

Specific Objectives:

1. To determine prevalence and correlates of helminth co-infection in HIV-1 infected individuals in various clinics in or around Nairobi, Kenya. Baseline demographic, socio-economic and geographic data such as age, gender, income, education, occupation and place of residence will be collected and compared to determine what factors influence the prevalence of helminth infection in patients with HIV-1.

2. To determine if treatment of intestinal helminths in HIV-1 infected adults can delay disease progression, as measured by CD4 counts and HIV-1 RNA viral loads.

3. To determine if treatment of intestinal helminths in HIV-1 infected adults can reduce shedding of genital HIV-1 and impact transmission.

DESIGNS AND METHODOLOGY

Study Site

The study will be conducted in Nairobi, Kenya. The University of Washington has a history of collaborative research with the Kenya Medical Research Institute, the University of Nairobi, and Kenyatta National Hospital; all leading academic institutions in Kenya. Enrollment in the randomized clinical trial will take place in several clinics in Kenya whyich may include clinics in Kibera, the KNH Comprehensive Care Clinic, KEMRI, Kiambu District Hospital, Homa Bay, Kerugoya, Coptic Hope Clinic, Mbagathi District Hospital, Thika District Hospital, and The Comprehensive Care and Research Clinic at Kilifi District Hospital (CGMR-C).

Study Populations

Individuals who are 18 years or older, who plan to reside within 25 kilometers of the study clinic site for at least 3 months after initiation of treatment and who are interested in study participation will be enrolled after written informed consent is obtained. Subjects must also meet the following inclusion/exclusion criteria;

Inclusion Criteria

- Participants must not be or have been on highly active antiretroviral therapy.
- Participants must have CD4 count >250 cells/mm^3 in order to be enrolled in the randomized controlled trial. Patients with lower CD4 counts may be screened but will not be randomized.
- Participants must be at least 18 years of age.
- Participants must be able and willing to participate and give written informed consent.
- Participants must be able and willing to return for the scheduled follow-up visits
- In addition, in order to be included in the treatment phase of the study, patients must have at least one stool specimen positive for a soil transmitted helminth.

Exclusion Criteria

- Participants who have received treatment for helminth infection in the past 6 months (by self report or chart review)
- Participants must not be pregnant at the time of treatment (by urine HCG testing).
- Participants who present with other serious co-morbidities such as severe anaemia, malaria or tuberculosis

Sample Size

Sample size determination – Assuming a power of 80% and alpha of 0.05, we have calculated that 100 individuals would be needed in each arm to detect a difference in CD4 count of 40 cells/mm^3 following anti-helminth therapy (SD of 200 cells/mm^3).

Study design
Specific Aim 1: observational cross-sectional study
Specific Aim 2: randomized clinical trial

Procedures

Summary: This study will be conducted in two phases. Initially, 20 HIV-1 infected patients from each of several clinic sites will be screened by stool microscopy for the presence of helminth co-infection. These data will be used to determine the prevalence of infection at various sites and to compare baseline variables between groups as described above. These data will also help determine which study site will likely provide a population with sufficiently high prevalence in which to conduct the second phase of the study. This second phase will be a randomized double blind, placebo controlled clinical trial of anti-helminth treatment among HIV-1 and helminth infected adults. All patients will receive therapy for helminth infection either immediately or deferred (at twelve weeks post enrolment). As patients will be screened at enrolment for helminth infection, total prevalence will be calculated and determined for the entire screened population.

Pilot study to identify optimal trial site: Initially, we will conduct a pilot study to determine the prevalence of helminth co-infection in HIV-1 infected individuals in several clinic sites at different locations in or around Nairobi and some potentially in more rural sites elsewhere in Kenya. Twenty HIV-1 infected individuals will be enrolled at each clinic site and fresh stool obtained for microscopy. Individuals with helminth infections will either be offered enrolment in the treatment phase of the study described below (if proximate to the proposed site of the clinical trial study) or treated with open label albendazole 400 mg a day for three days and referred back to the clinic in which they receive regular care.

The study site that is the most feasible for the trial will be selected based on the data from the pilot study (specific aim 1) to be the base for larger scale screening to identify 200 HIV-1 infected helminth co-infected individuals. Specific factors that will be considered for site selection include helminth prevalence and ability to recruit HIV-1 infected helminth co-infected individuals. Depending on helminth prevalence this may require screening ~600 to 2000 individuals (10% to 30% estimated helminth prevalence).

Clinical Trial:

a) Screening for trial eligibility: At the selected clinical trial study site, HIV-1 infected individuals who meet inclusion and exclusion criteria will be informed about screening for stool helminths. A physical examination will be conducted on all prospective clients, and those found to have clinical pallor, or signs and symptoms of tuberculosis, malaria or malignancy will be excluded from the study and referred for appropriate medical management. Individuals with CD4 counts >250 cells/mm^3 will be eligible for study screening following written informed consent for study screening. For those individuals without available CD4 count data, patients will be screened if there is no evidence of WHO Stage II-IV disease. Patients who are subsequently found to have CD4 counts less than 250 cells/mm^3 will be referred for further HIV care at the referring clinic. The individual clinic sites will be notified of all CD4 count results within 14 days of testing. Baseline demographic and socio-economic data will be collected from these patients using standardized questionnaires. Stool samples will be collected and evaluated for ova and parasites. Participants who are found to have positive stool parasite examinations will be

informed about the clinical trial and those who consent to participate will be enrolled in the treatment phase of the study. Those who are found to be infected with helminths but who decline to participate in the treatment phase will be treated at no charge with a three day course of 400mg/day of albendazole by the study investigators. Those who are found to have negative stool specimens will not continue to the treatment phase. After screening exit (either due to lack of helminths or declining to participate), individuals will be referred back to the clinic in which they receive their usual care.

b) *Clinical trial:* HIV-1 infected helminth co-infected individuals who elect to participate in the randomized clinical trial will be enrolled following written informed consent. Those patients who are enrolled in the treatment phase will have blood collected for baseline plasma HIV-1 RNA levels and CD4 cells. Computer-generated random number allocation will be used to assign treatment group. The pharmacy at the clinic will be responsible for the determination of randomization group and distribution of albendazole and placebo. The pharmacy will also be responsible for maintaining records regarding treatment group. Treatment will be provided in the form of three 400 mg pills of albendazole or a three placebo pills identical in appearance (to be taken one pill per day for each of three consecutive days). Both placebo and albendazole will be provided in an identical appearing envelope. Treatment arms will consist of either initial therapy with albendazole (400 mg a day for three days) or an initial three days of placebo pills. All patients will be required to visit the clinic eight weeks after enrollment. At this twelve-week visit, stool samples will again be collected for evaluation of helminth infection. Plasma CD4 count and HIV-1 RNA level measurements will be repeated at this twelve-week visit. All patients who received placebo initially as well as all patients with evidence of helminth infection will be treated with open-label albendazole therapy regardless of initial treatment arm.

At enrollment and the twelve-week visit, lymphocytes will also be isolated from serum samples for assessment of immune activation markers. Genital HIV-1 RNA levels will be compared in 100 individuals randomized to immediate versus deferred antihelminthics at baseline and at the 3-month visit (prior to receiving deferred therapy). This will provide data quantifying the potential effect of anti-helminthic treatment on genital HIV-1 RNA. Consenting adults will have genital swabs collected (semen or cervical/vaginal) and placed in freezing media prior to cryopreservation and transport to Fred Hutchinson Cancer Research Center for HIV-1 RNA assays.

Patients who do not follow-up as directed will be contacted by the administrative support team at their homes in order to determine the reason for each loss to follow-up. Any adverse events during the treatment period will be reported and managed accordingly at the treatment clinic.

Laboratory

CD4 measurement will be assessed by FACSCalibur in Nairobi, Kenya.

Stool microscopy will be performed by technicians with training and certification in the differentiation and quantification of stool helminth species. Testing will be done using conventional microscopic techniques.

HIV-1 RNA levels will be quantified at the Julie Overbaugh's laboratory at Fred Hutchinson Cancer Research Center in Seattle. Below is an explanation of the reasons for preference of this laboratory site:

1. Research collaborations have involved long-standing relationships with the University of Nairobi, KEMRI, and KNH, and University of Washington and the Fred Hutchinson Cancer Research Center. This collaboration has conducted studies in HIV infected individuals in Kenya for many years. HIV-1 RNA assays for these studies have been done at the laboratory of Dr. Julie Overbaugh in Seattle, USA. Dr.

Overbaugh has made significant efforts to transfer technology for HIV-1 DNA filter paper assays and HIV-1 RNA assays for research studies. We are optimistic that DNA filter paper assays and potentially HIV-1 resistance assays will be conducted in Nairobi within 2 years.

2. Over the past many years, Dr. Overbaugh's laboratory has taken care to delineate subtype-related issues to optimize measurement of HIV-1 RNA in Kenya with a non-commercial Gen-Probe TMA (transcription-mediated assay) specifically tailored for subtypes A, D, and C from Kenyan isolates. Thus, it is useful to correlate the proposed study to other large-scale studies in Nairobi conducted by our research team.
3. Available funding for this study will not be sufficient to measure viral RNA in even 60 individuals at 2 time-points if these measurements were done locally (cost per assay in Nairobi ~4-5 times the cost at Dr. Overbaugh's laboratory). Under the current budget constraints we would likely be limited to a small subgroup analysis of 30 patients.

   Given the improved design of the study with an increased sample size including 200 individuals, measuring HIV-1 RNA in all participants would significantly strengthen the study and increase the potential that significant effects on viral decline would be found.

Immune activation markers will be assessed using flow cytometry available at the University of Nairobi. Antibodies to relevant cell surface molecules, in combination with either anti-CD4 or -CD8 will be used for a measure of global T cell activation. Numbers of $CD4^+$ and $CD8^+$ T cells expressing the nuclear antigen, Ki67, will be used as a marker for ex vivo proliferating cells. Naïve and memory subsets will be determined from $CD4^+$ and $CD8^+$ T cell populations by staining patterns for differentiation markers CD45 and CD27: $CD45RA^+$ $CD27^+$ (naïve), $CD45RA^-$ $CD27^+$ (memory), $CD45RA^+CD27^-$ (effector) and $CD45RA^-$ $CD27^-$ (memory/effector), CD-38, and HLA-DR. HIV-1 specific responses (CD8 interferon gamma and IL-2) will be determined using Elispot and intracellular cytokine staining (ICS) in a subset of 20 individuals using cryopreserved PBMC specimens. Flow-Jo software will be utilized to quantify results.

Data Management

Data Entry and Storage: All data will be collected on preprinted forms that are able to be scanned directly or data entered into a software data management program (Teleform SPSS or ACCESS). Data will be collected at enrollment and at each follow up visit on these forms. Following collection, the data forms will be scanned into the database as described above. Original data forms will be stored in the study offices with access restricted to study investigators. Following completion of all data collection, the data forms will be archived. At 1-year following completion of the study, identifiers will be removed from the data. Data will be the property or KEMRI and the University of Washington.

Data verification: A data clerk from KEMRI will be employed to hand verify that all data is completed accurately and that the computerized scanned data is comparable to the paper forms. Data will be cleaned by the Fellow Investigator and the data clerk and at that time a second verification of accuracy will be performed.

Data Analysis:

Aim 1: Prevalence of helminth co-infection will be determined in the total screened population by individual, by clinic and by cohort. To determine correlates of helminth co-infection, mean CD4 count, age and $\log_{10}$ RNA levels will be compared in individuals with and without helminth co-infections using t-tests. Dichotomous variables (socio-demographic) will be compared with Chi square tests in individuals with and without helminth co-infection. Similar analyses will be conducted to determine correlates of re-infection and eradication.

Aim 2: All analyses will be intent-to-treat. To assess the success of randomization we will compare baseline characteristics between the 2 randomization groups. To determine the effect of antihelminth treatment on disease progression markers, we will compare month one measurements of mean CD4 count, $\log_{10}$ plasma HIV-1 RNA, and percentage of cells with specified immune activation markers in the 2 study arms using t-tests or non-parametric testing. In addition, prevalence of helminth co-infection will be compared in the 2 study arms using Chi-square tests. To determine the effect of antihelminth treatment on infectivity, we will compare enrollment measurements of mean genital HIV-1 RNA levels with the 12 week follow up measurements.

Time Schedule

Total period of project is 12 months

We anticipate a 2-month preparation period followed by 7 months of recruitment and follow-up. Laboratory and data analysis will take 2 months and manuscript preparation will take 1 month.

Ethical Considerations:

***Ethical approval*- Study approvals will be obtained from the University of Washington and the KEMRI ethical review boards.**

Benefits: This study has been designed to address several areas of major public health significance for HIV-1 infected individuals in resource poor settings. If we are able to show that treatment of helminth co-infection in HIV-1 infected individuals delays immunosuppression, millions of HIV infected individuals in resource poor settings may benefit. Clients who have helminth infections will benefit from free treatment and examination given to them.

Voluntary Participation: All subjects will provide written informed consent prior to study enrolment. Consent forms will be made available in Swahili as well as in English. All patients will have the opportunity to have the forms explained to them and to ask questions of the investigator prior to study enrolment. Patients will be informed of their right to withdraw consent at any time. Since the participants will spend extra time to participate in the study, they will receive compensation for transport, but no other compensation will be provided to them.

Risks: Patients will be informed of all potential risks. The first risk may be associated with use of the study medication, albendazole. Albendazole is a benzimidazole carbamate derivative with activity against most nematodes and some other worms. Albendazole is thought to inhibit cytoplasmic microtubules in the worm's intestinal tract leading to decreased glucose uptake and depletion of glycogen stores in the worm. Three 400 mg doses of albendazole has efficacy against whipworm, hookworm and roundworm infections, all of

which we expect to be prevalent in this cohort. A recent randomized controlled trial of various albendazole dosing regimens showed that a single 400mg dose of albendazole was associated with a significantly lower rate of cure (23%) compared to a three day regimen (67%). In this study, reduction in the number of eggs/gm of feces with a single 400mg dose was 96.8% compared to 99.7% with the three day regimen[13]. Albendazole is minimally absorbed from the gastrointestinal tract and has minimal side-effects. Patients will be screened for helminth infection at enrolment and subsequently randomized to immediate versus deferred (twelve weeks later) treatment of intestinal helminth infection. Patients with obvious clinical signs or symptoms of anaemia, significant diarrhoea or abdominal pain will not be enrolled. A twelve week treatment delay in those patients who are asymptomatically infected but do not receive active drug is not anticipated to have any adverse health consequences. Neither routine screening of asymptomatic helminth infection nor empiric anti-helminth therapy is currently conducted in adults in Kenya. Patients will have blood withdrawals at enrolment and during follow-up. This may be associated with bleeding or swelling at the injection site. Precautions will be taken to avoid bleeding by immediate application of a pack and pressure at the injection sites.

A subset of participants (100 individuals) will be included in the study of genital HIV-1 RNA levels. This will require collection of a semen sample from male participants and collection of vaginal and/or cervical samples from female participants. Participants may find this embarrassing. Female participants will undergo a pelvic examination for collection which may be uncomfortable.

Confidentiality: Patients will be assigned a study number at enrollment. This number will be used to identify patients for all matters related to data analysis. The forms linking patient names and demographic information to particular ID numbers will be kept locked in a file at the office of the Fellow Investigator.

Expected application of result:

We anticipate that the proposed study would lay the foundation to develop a series of clinical epidemiology studies to determine the role that common helminth co-infections play in HIV-1 pathogenesis, progression and transmission. The proposed study will generate data to be used in the design of a large, randomized, double blind study evaluating the effect of prophylactic anti-helminth treatment in HIV-1 infected individuals in helminth-endemic areas. In order to design a large clinical trial to determine the long-term effect of antihelminth therapy, it is important to have data on current urban prevalence of helminth infections in HIV-1 infected individuals as well as on the short-term effect of helminth eradication. These data will enable informed projections specifically regarding estimated efficacy and necessary sample size.

References:

[1] 2004 report on the global AIDS epidemic : 4th global report. UNAIDS

[2] Fincham JE, Markus MB, Adams VJ. Could control of soil-transmitted helminthic infection influence the HIV/AIDS pandemic. Acta Trop. 2003 May;86(2-3):315-33.

[3] Elliott AM, Mawa PA, Joseph S, Namujju PB, Kizza M, Nakiyingi JS, et al. Associations between helminth infection and CD4+ T cell count, viral load and cytokine responses in HIV-1-infected Ugandan adults. Trans R Soc Trop Med Hyg. 2003 Jan-Feb;97(1):103-8.

[4] Bentwich Z, Weisman Z, Moroz C, Bar-Yehuda S, et al. Immune dysregulation in Ethiopian immigrants in Israel: relevance to helminth infections. Clin Exp Immunol **1996**;103:239-43.

[5] Kassu A, Tsegaye A, Wolday D, Petros B, et al. Role of incidental and/or cured intestinal parasitic infections on profile of CD4 and CD8 T cell subsets and activation status in HIV-1 infected and uninfected adult Ethiopians. Clin Exp Immunol **2003**;132:113-119.

[6]Elliott AM, Kyosiimire J, Quigley MA, Nakiyingi J et al. Eosinophilia and progression to active tuberculosis in HIV-1-infected Ugandans. Trans R Soc Trop Med Hyg. 2003 Jul-Aug;97(4):477-80.

[7] Olsen A. The proportion of helminth infections in a community in Western Kenya which would be treated by mass chemotherapy of school children. Trans R Soc Trop Med Hyg **1998**;92:144-8.

[8] Wolday D, Mayaan S, Mariam ZG, Berhe N, et al. Treatment of intestinal worms is associated with decreased HIV plasma viral load. J Acquir Imm Defic Syndr **2002**;31:56-62.

[9]Lawn SD, Karanja DM, Mwinzia P, Andove J, et al. The effect of treatment of schistosomiasis on blood plasma HIV-1 RNA concentration in coinfected individuals. AIDS **2000**;14:2437-43.

[12] Quinn TC et al. Viral load and heterosexual transmission of human immunodeficiency virus type 1. N Engl J Med 2000;342: 921-929.

[11]Adams VJ, Lombard CJ, Dhansay MA, Markus MB, et al. .Efficacy of albendazole against the whipworm trichuris trichiura--a randomised, controlled trial.S Afr Med J. 2004 Dec;94(12):972-6.
